# Dissecting the Genetic Architecture of Cystatin C in Diversity Outbred Mice

**DOI:** 10.1534/g3.120.401275

**Published:** 2020-05-28

**Authors:** M. Nazmul Huda, Melissa VerHague, Jody Albright, Tangi Smallwood, Timothy A. Bell, Excel Que, Darla R. Miller, Baback Roshanravan, Hooman Allayee, Fernando Pardo Manuel de Villena, Brian J. Bennett

**Affiliations:** * Obesity and Metabolism Research Unit, Western Human Nutrition Research Center, USDA, ARS, Davis, California 95616,; ^†^ Department of Nutrition, University of California Davis, California 95616,; ^‡^ Nutrition Research Institute, University of North Carolina Kannapolis, North Carolina 28081,; ^§^ Department of Genetics, University of North Carolina at Chapel Hill, North Carolina 27599,; ** Department of Medicine, Division of Nephrology, University of California, Davis, Davis, California 95616, and; ^††^ Departments of Preventive Medicine and Biochemistry & Molecular Medicine, Keck School of Medicine, University of Southern California, Los Angeles California 90033

**Keywords:** Quantitative trait loci, Multi parental models, Cystatin C, Kidney biomarkers, Type-I interferon signalling pathway, Multiparent Advanced Generation Inter-Cross (MAGIC), multiparental populations, MPP

## Abstract

Plasma concentration of Cystatin C (CysC) level is a biomarker of glomerular filtration rate in the kidney. We use a Systems Genetics approach to investigate the genetic determinants of plasma CysC concentration. To do so we perform Quantitative Trait Loci (QTL) and expression QTL (eQTL) analysis of 120 Diversity Outbred (DO) female mice, 56 weeks of age. We performed network analysis of kidney gene expression to determine if the gene modules with common functions are associated with kidney biomarkers of chronic kidney diseases. Our data demonstrates that plasma concentrations and kidney mRNA levels of CysC are associated with genetic variation and are transcriptionally coregulated by immune genes. Specifically, Type-I interferon signaling genes are coexpressed with *Cst3* mRNA levels and associated with CysC concentrations in plasma. Our findings demonstrate the complex control of CysC by genetic polymorphisms and inflammatory pathways.

The kidney is a complex organ responsible for excretion (Finco 1997), secretion (Davis *et al.* 1961), reabsorption (Lemann *et al.* 1970), and activating vitamin D (Fraser and Kodicek 1973). The gold standard for assessing kidney function is the glomerular filtration rate (GFR), but it is difficult to measure with precision. Therefore, GFR is often estimated from circulating creatinine (Ferguson *et al.* 2015). Creatinine is an amino acid derivative, released by muscle, and freely filtered by the kidney glomerulus (Narayanan and Appleton 1980). However, the level of plasma creatinine is influenced by a number of factors, including: diet, muscle mass, medication, chronic illness, age, sex, and race, limiting its accuracy to represent true GFR (Stevens *et al.* 2006). An alternative to creatinine is Cystatin C (CysC), often used in research as the basis for estimating glomerular filtration rate. CysC is produced by all mammalian cells, secreted into the blood, filtered through the glomerulus, and catabolized by tubular cells (Inker and Okparavero 2011). Plasma CysC had been found to be unaltered by age, sex, race, and metabolic disorders and was proposed as a clinical biomarker for GFR (Filler *et al.* 2005; Newman *et al.* 1995). Several clinical trials reported CysC as a superior marker compared to creatinine (Plebani *et al.* 1998; Kyhse-Andersen *et al.* 1994; Harmoinen *et al.* 1999) while a few others could not show a significant difference between CysC and creatinine (Donadio *et al.* 2001; Oddoze *et al.* 2001). Although CysC is not influenced by physiological factors, several recent studies (Köttgen *et al.* 2010; Köttgen *et al.* 2009; Hwang *et al.* 2007) have found associations between SNPs near the Cystatin C gene (*Cst3*) and plasma CysC levels or CysC-based estimated glomerular filtration rate (eGFRcys). How these genetic variants relate to plasma CysC protein level or its mRNA level remains to be determined.

In the current study, we focus on the genetic determination of CysC protein and mRNA levels using a “Systems Genetics” approach, which is useful for the discovery of genes and pathways associated with a reduced kidney function (Keller *et al.* 2012). We also perform co-expression analysis and expression QTL (eQTL) analysis to further investigate the control of CysC levels using the Diversity Outbred (DO) population, which are derived from eight founder strains (A/J, C57BL/6J, 129S1/SvImJ, NOD/ShiLtJ, NZO/HiLtJ, CAST/EiJ, PWK/PhJ, and WSB/EiJ). Since the DO is a highly recombinant population with immense genetic and phenotypic variation (Svenson *et al.* 2012; Smallwood *et al.* 2014), DO mice have been successfully used in studies focused on the genetic response to environmental toxin exposure and diseases (French *et al.* 2015; Tyler *et al.* 2017). The DO mice capture the same set of allelic variants as the eight founder strains, and their genetic backgrounds are well-studied, which make them an excellent model for researching the genetic susceptibility to disease. In this study, we focus on the genetic variation associated with CysC gene expression and plasma CysC concentration in DO mice. We then performed co-expression network analysis to identify gene modules associated with CysC and identified two gene modules associated with CysC levels.

## Materials and Methods

### Study design and sample collection

Female diversity outbred (DO) mice (n = 120; J: DO; G16; stock number 009376) were obtained from the Jackson Laboratory (Bar Harbor, ME) at 4 weeks of age. These mice were used to generate progeny for another study (Didion *et al.* 2015) and then aged. At 56 weeks of age, all mice were injected intraperitoneally with a volume of sterile isotonic saline equivalent to 10% of their body weight and urine was collected in a chilled metabolic cage system (Hatteras Inc, NC). On the following day, mice were fasted for 4 hr, plasma was collected from the retro-orbital sinus into EDTA-containing microtubes and centrifuged. Mice were euthanized, dissected, and tissue samples were snap frozen in liquid nitrogen. Biological samples were stored at -80° until assayed. All procedures were approved by the IACUC at UNC, Chapel Hill (IACUC Protocol Number 13-103).

### Plasma and urine biochemical assays

Plasma CysC was measured by a commercially available quantitative sandwich ELISA kit (R&D Systems, MN, USA) for mice according to the manufacturer’s instructions. In brief, plasma samples were diluted 200-fold and incubated in a 96 well microplate pre-coated with CysC specific antibody and CysC concentration was determined from the color intensity of oxidized Tetramethylbenzidine (TMB) measured at 450 nm. Urinary total protein, creatinine and plasma blood urea nitrogen were measured by COBAS INTEGRA 400 plus analyzer (Roche Diagnostics, Rotkreuz, Switzerland). To measure urinary Na^+^, samples were diluted 1500-fold with 1.0% v/v trace metal free nitric acid and analyzed by using a Varian VISTA AX CCD Simultaneous Inductively Coupled Plasma Atomic Emission Spectroscopy (Varian, CA, USA). Standards Na^+^ for Inductively Coupled Plasma Mass Spectrometry (Spex CertiPrep, NJ, USA) was used to make standards ranging from 0.05 ppm to 5.0 ppm. Certified urine controls (Seronorm, Stasjonsveien, Norway) and sample pool controls were used to check the accuracy of the assay.

### Kidney mRNA microarray

Total RNA was extracted from about 25 mg of kidney tissue using automated instrumentation (Maxwell 16 Tissue LEV Total RNA Purification Kit, Promega). RNA concentration was measured by fluorometry (Picogreen Life Technologies), and RNA quality was verified using a microfluidics platform (Bioanalyzer, Agilent Technologies). 95 RNA samples were chosen for microarray analysis and 1 sample was run in duplicate as a control. RNA was hybridized to Affymetrix Mouse Gene 2.1 ST 96-Array Plate using a GeneTitan instrument from Affymetrix according to the manufacturer’s protocols. We used the robust multiarray average method (RMA) implemented in the Affymetrix gene expression console with default settings (median polish and sketch-quantile normalization) to estimate the normalized expression levels of transcripts. All probes containing known single nucleotide polymorphisms (SNPs) from the eight founder inbred mouse strains of the DO mouse population were masked (165,204 probes) during normalization by downloading the SNPs from the Sanger sequencing website (http://www.sanger.ac.uk/science/data/mouse-genomes-project) and overlapping them with probe sequences. The data are available at NCBI Gene Expression Omnibus (GEO) database under the accession ID GSE122061.

### Genotyping

DNA was extracted and purified from tail samples using Qiagen DNeasy kit (Qiagen, MD, USA) according to the manufacturer’s instructions. Genotyping was performed using the Mega Mouse Universal Genotyping Array (MegaMUGA) by GeneSeek (Neogen, Lansing, MI) (Welsh *et al.* 2012). The MegaMUGA array is built on the Illumina Infinium platform and contains 77,800 SNP markers that are distributed throughout the genome at an average spacing of 33 Kb. Genotypes of DO mice are accessible through UNC’s Mutant Mouse Resource and Research Centers website (https://www.med.unc.edu/mmrrc/genotypes/). We estimated heritability (*h^2^*) from allele probability using a linear mixed model in R package QTL2 version 0.18 (Broman *et al.* 2019).

### QTL mapping

QTL mapping and Genome-wide association (GWAS) analysis were performed using the R package QTL2 (Broman *et al.* 2019) on the University of California, Davis’ high-performance cluster computing system, which has 6,752 CPUs with 64 GB – 1 TB of RAM on each node. Genotype probability was calculated from the allele calls. The probability of a founder SNP haplotype was calculated from genotype probabilities. QTL mapping was carried out by regressing the phenotypes on the founder haplotypes with an adjustment for kinship matrix as a random effect of the linear mixed effect model in the QTL2 “scan1” and “scan1perm” functions. Kinship matrix is a measure of genetic similarity among individuals used to control for the random polygenic effect in genome scanning. In this study, we calculated a matrix of the proportion of matching alleles per chromosome using the leave out one chromosome at a time (type = “loco”) method in the QTL2 R package. Candidate genes were identified by position based on the Wellcome Trust Sanger mouse genomes database, www.sanger.ac.uk, release 1303 based on genome assembly GRCm38/mm10 (Yalcin *et al.* 2011). QTL support intervals were defined by the 95% Bayesian Credible Interval (BCI), calculated by normalizing the area under the QTL curve on a given chromosome (Chen and Kendziorski 2007). We used the Best Linear Unbiased Predictors (BLUPs) model (Robinson 1991) in the QTL2 package to estimate the allelic contributions of the 8 founder strains to the significant QTL in which the founder allelic effect was identified using a regression of the phenotype on the founder genotype probabilities at the locus. The mapping statistic reported as a log of the odds ratio (LOD). The significance LOD thresholds at *P* < 0.05 for each kidney biomarker and gene probeset were determined by performing 1,000 permutations of genome-wide scans by shuffling genotype data in relation to phenotype data to generate a null distribution from the maximum LOD score (Churchill and Doerge 1994). A QTL or eQTL was considered significant when the LOD score for the phenotype is above the permutation LOD threshold at *P* < 0.05 for that particular phenotype. Instead of estimating a single global LOD threshold from permutation testing of a randomly selected subset of genes, we performed permutation testing for each annotated transcript cluster ID to determine its individual LOD threshold. A single LOD threshold for all transcript cluster IDs allows variation of the significant p-value cut off across transcript cluster ID, leading to possible increases in both Type –I error for some transcript cluster ID and type-II error for others (Figure S1). To accomplish > 23,000,000 genome scans, we utilized a high-performance cluster computing system, which allowed us to perform the parallelization of CPU for computationally intensive permutation genome scans to empirically determine the significance threshold for each annotated transcript cluster ID on the microarray. An eQTL for microarray data were considered “*cis*” when a transcript cluster ID was located at the same genomic position (within a ± 2Mb interval) of the probe (Bennett *et al.* 2010). We compared the single empirical LOD threshold-based eQTL results to the individual threshold method and identified 274 transcript cluster ID with Type-II error or false negative. Similarly, the single empirical LOD based analysis incorrectly identified 1,495 non-significant eQTLs as significant (Type-I error or false positive). Therefore, in our study, we found a single empirical LOD threshold-based analysis had 49.5% Type-I error and 9.1% Type-II error (Figure S1C).

### Weighted Gene Correlation Network Analysis

Co-occurrence network analysis was performed by using R package WGCNA-Weighted Gene Correlation Network Analysis (Langfelder and Horvath 2008) version 1.66. Gene expression data were available for 95 samples. A total of 23,612 transcript cluster IDs were filtered to 8,045 those were expressed above robust multi-array average (RMA) value of 6 in more than 87.5% of the samples (Aylor *et al.* 2011). 4 samples were identified as outliers (Figure S2A) and removed, resulting in 91 samples for gene module analysis. A soft threshold approach was used with a power of 4 (based on scales free topology, Figure S2B and S2C) in a WGCNA default unsigned network with dynamic tree cutting (deep split = 2) and a min Module Size = 15 as parameters for the dynamic tree cut function (Langfelder *et al.* 2007). The module eigengene, defined as the first principle component of a module’s gene expression matrix, was used to calculate the Spearman correlation between a module and kidney biomarkers. Gene network modules were visualized using Cytoscape 3.7.1 (Shannon *et al.* 2003).

### Functional annotation

We performed gene ontology (GO) and Kyoto Encyclopedia of Genes and Genomes (KEGG) pathway enrichment analysis on each module using Enrichr (Chen *et al.* 2013) with “GO biological process 2018” and “KEGG 2019 mouse” dataset, respectively, to determine if any gene set in the modules were associated with shared functional annotations or biochemical pathway. For each term in the gene-set library, the rank-based ranking of each of the GO term was derived from running Fisher’s exact test for many random gene sets to compute a mean rank and standard deviation from the expected rank and then z-score was calculated to assess the deviation from the expected rank of the enriched GO term. The combined score for the enriched GO term was computed by multiplying the z-score and -log of the p-value of the Fisher exact test. Gene annotation for tissue-specific expression was performed using several databases including: DAVID (Huang *et al.* 2009) and BioGPS Mouse Cell Type and Tissue Gene Expression dataset (Wu *et al.* 2013). The human GWAS data at the CysC locus were obtained from the European population (Pattaro *et al.* 2016) and was queried for LocusZoom (Pruim *et al.* 2010) plotting. The effect of SNP and indel variation on protein function was determined by a web-based tool Protein Variation Effect Analyzer -PROVEAN (Choi and Chan 2015). *In silico* transcription factor (TF) binding site prediction was performed by using CiiiDER (Gearing *et al.* 2019) with JASPAR 2020 motif database (Khan *et al.* 2018). The functional relevance of SNPs located within the predicted transcription motif was estimated by using R package for tRAP (Thomas-Chollier *et al.* 2011).

### Quantitative PCR

To validate the kidney genes expression we utilized archived kidney samples from a strain survey of DO/CC Progenitor mice (O’Connor *et al.* 2014) in which the mice were perfused prior to tissue collection. Total RNA was isolated using MagMAX mirVana Total RNA Isolation Kit (Thermo Fisher, MA, USA) and cDNA was synthesized using High-Capacity cDNA Reverse Transcription Kit (Thermo Fisher, MA, USA) according to the manufacturer’s recommendation. The qPCR was performed using the primer listed in the Table S1 using PowerUp SYBR Green Master Mix (Thermo Fisher, MA, USA) on QuantStudio 6 and 7 Flex Real-Time PCR Systems (Thermo Fisher, MA, USA). PCR was run in triplicate and relative gene expression was determined using an efficiency corrected method, and efficiency was determined from a 2-log serial dilutions standard curve made from cDNA pooled from all samples (Bennett *et al.* 2013) and normalized by GAPDH expression.

### Other statistical analyses for the phenotype data

Clinical phenotype data for DO mice were checked for normality. Non-normally distributed data were transformed using log10, Box-Cox power, or rank-normal functions before any statistical analysis. The variables with Shapiro-Wilk “W” value >0.95 were considered as normally distributed. The correlation between CysC and variables indicating kidney biomarkers were determined by Spearman correlation. Regression analysis involving CysC was adjusted for body weight as a confounding factor. Data are presented as means ± SD unless otherwise indicated. Statistical analysis was performed using R 3.6.1 for windows release (R Core 2019). Correlation p-value was adjusted for multiple comparisons using “Benjamini & Hochberg” (Benjamini and Hochberg 1995), and an adjusted p-value <0.05 was considered significant for all analyses unless stated otherwise.

### Data availability

Microarray gene expression data are available at GEO with the accession ID GSE122061. Genotypes of DO mice are accessible through UNC’s MMRRC website (https://www.med.unc.edu/mmrrc/genotypes/). Further information is available from the corresponding authors if required. This study was approved by the IACUC at UNC, Chapel Hill (IACUC Protocol Number 13-103). DO mouse kidney RNASeq (DO-RNASeq) data are available at the NCBI’s Gene Expression Omnibus (GEO) with the accession ID GSE121330 and also at Dr. Churchill’s laboratory web site (https://churchilllab.jax.org/qtlviewer/JAC/DOKidney). The C57Bl/6J mouse RNASeq (B6-RNASeq) data (Söllner *et al.* 2017) is available at https://www.ncbi.nlm.nih.gov/bioproject/?term=PRJNA375882 and EBI under the Array Express ID: E-MTAB-6081; https://www.ebi.ac.uk/arrayexpress/experiments/E-MTAB-6081/. Supplemental material available at figshare: https://doi.org/10.25387/g3.10307972.

## Results

### Characteristics of the study DO mice population

The mean body weight of the mice was 32.4 ± 7.4g with a range from 19.0 to 58.5g; a variation of approximately 23% among our DO study population (Table 1). Plasma CysC had similar variation as bodyweight, approximately 25% variation, with a mean concentration of 535.5 ± 137.5 ng/mL and ranging from 260 to 922 ng/ml. Although both body weight and plasma CysC had similar variations within the population of mice they were not significantly correlated with each other (r = 0.028, *P* = 0.80).

**Table 1:** Characteristics of the study mice

Characteristics	*n**^a^*	*Mean ± SD*	*Median (25^th^*, *75^th^)*	*Correlation with Cystatin C*		*Correlation with Cystatin C (Cst3) mRNA**^b^*	
				*r*	*p*	*r*	*p*
Body weight, *g*	120	32.4 ± 7.4	31.5 (26.3, 37.3)	−0.014	0.88	0.028	0.80
Plasma cystatin C, ng/mL	109	535.5 ± 137.5	515.1 (427.7, 629.5)	1.000	NA	0.500	**1.22 x 10^−06^**
Blood urea nitrogen, mg/dL	120	14.9 ± 5.0	14.3 (11.9, 16.7)	−0.079	0.41	−0.097	0.38
Urine pH	111	6.6 ± 0.8	6.5 (6.0, 7.2)	−0.062	0.54	−0.085	0.44
Urine Osmolality, mOsm/kg of water	117	534.8 ± 201.2	491 (400, 625)	0.181	*0.062*	0.219	0.046
Urine volume, µL	120	826.4 ± 651.4	745.0 (285.0, 1210.0)	−0.126	0.19	−0.007	0.95
Urinary protein, mg/L	83	369.4 ± 321.7	255.6 (105.2, 570.4)	0.176	0.12	0.177	0.16
Urinary creatinine, mmol/L	83	0.3 ± 0.2	0.3 (0.3, 0.5)	0.183	0.11	0.228	0.07
Urinary protein: creatinine, mg/mmol	83	2080 ± 6530	789 (349, 1400)	0.121	0.29	0.112	0.37
Urinary total Na, ng	92	2415 ± 1526	2060 (1296, 3380)	0.006	0.95	0.121	0.31
Na excretion rate, ng/h	92	1208 ± 763	1030 (648, 1690)	−0.003	0.98	0.113	0.34
Urinary Na: creatinine, ng/mmol	82	10.3 ± 16.7	6.8 (4.8, 10.6)	−0.122	0.29	−0.128	0.31
Urinary total K, ng	92	1002 ± 698	876 (514, 1326)	0.045	0.68	0.115	0.34
K excretion rate, ng/h	92	501 ± 349	438 (257, 663)	0.020	0.85	0.078	0.52
Urinary K: creatinine, ng/mmol	82	3.7 ± 5.0	2.9 (2.1, 3.8)	−0.202	*0.08*	−0.249	**0.045**

a Number represents the number of successful mouse phenotype observes depending on the availability of the sample.

b Gene expression data were available for 95 mice.

We also measured levels of the following urinary analytes: creatinine, total protein, protein: creatinine ratio, total sodium, sodium excretion rate, sodium: creatinine ratio, total potassium, potassium excretion rate, and potassium: creatinine ratio. Additionally, blood urea nitrogen was measured. We observed a significant variation within urinary creatinine, urinary protein, urinary total protein: creatinine, urinary total sodium, urinary sodium excretion rate, and urinary sodium: creatinine (Table 1). To better assess how these urinary markers are related to renal function, we correlated plasma CysC and cystatin C (*Cst3*) gene mRNA expression levels with each urinary analyte. Plasma CysC did not show any significant correlation with other blood or urinary kidney biomarkers (Table 1). *Cst3* expression level was significantly correlated with CysC (r = 0.498, *P* = 1.22 × 10^−06^) and Urinary K: creatinine (r = -0.249, *P* = 0.045; Table 1).

### Quantitative trait locus (QTL) analysis of plasma and urinary traits demonstrate that plasma CysC concentration is under genetic determination in the DO

**Figure 1:**
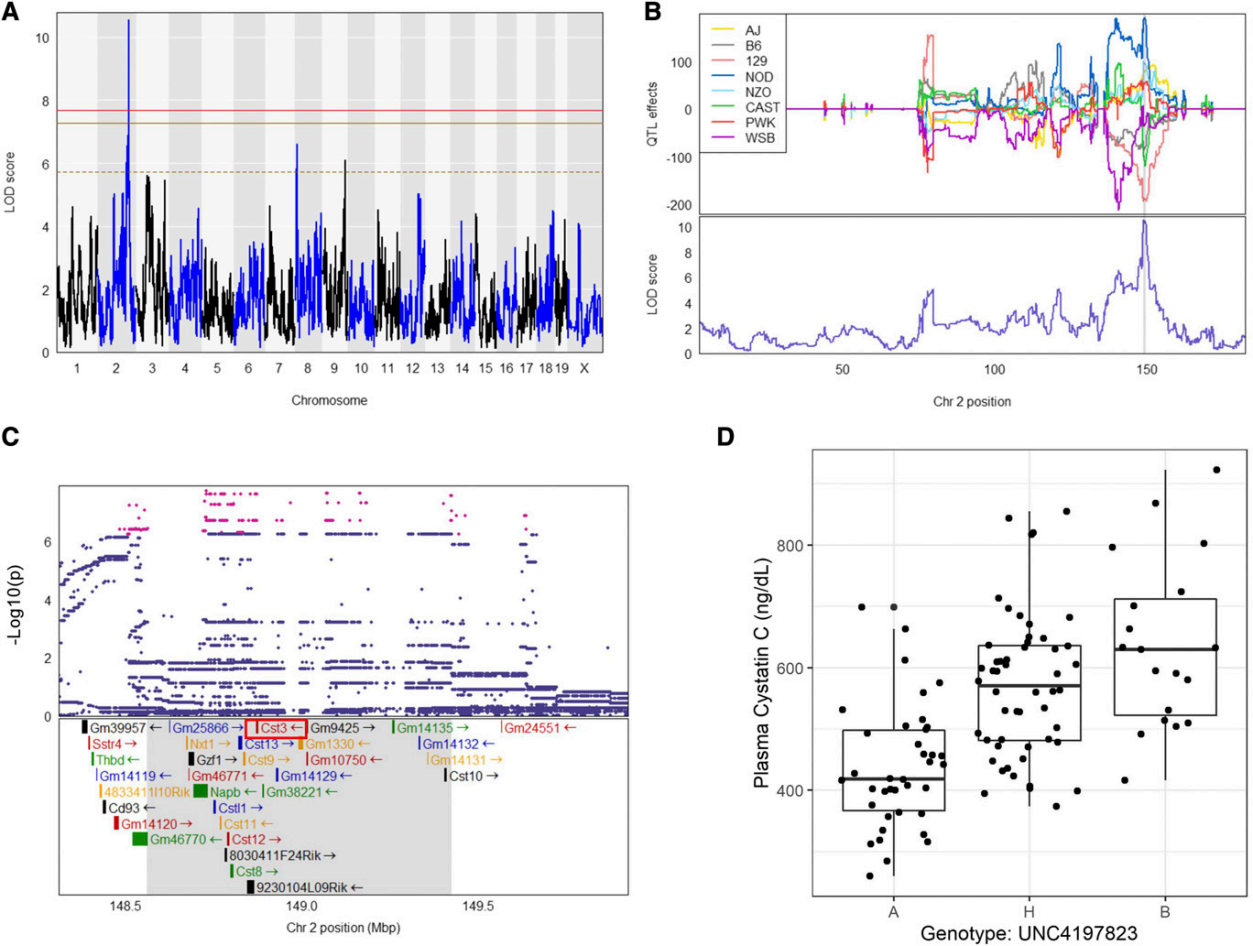
Plasma cystatin C QTL. (A) Genome scan of plasma CysC level. Red, solid golden, and broken golden lines show permutation-derived (N = 1000) significance thresholds at *P* < 0.05, *P* < 0.10, and *P* < 0.63, respectively. (B) The Best Linear Unbiased Predictors (BLUPs) coefficient plot of eight founder mice strains to the CysC QTL (top). Color represents the eight founder mice strains as indicated. The bottom portion represents the LOD score for plasma CysC on the chromosome 2. (C) Zoomed view of the peak position with an additive SNP model and the known genes in that region. Red dots indicate the significant SNPs at *P* < 0.05 (D) Genotype by phenotype plot of the top SNP located at the peak LOD for plasma CysC concentration. Genotype: A – AA, B – GG and H – AG. Shaded areas on the figure B and C represent approximate 95% Bayesian credible interval.

We first assessed the heritability (*h^2^*) of plasma CysC which was 47.5% in our DO population, indicating a significant portion, nearly 50%, of the variation in plasma CysC is genetic. To identify if there are specific loci regulating CysC levels, we next performed QTL analysis. We identified a single locus regulating plasma CysC on Chromosome 2 at position 148.7 Mb with a logarithm of the odds ratio (LOD) score of 10.6 which exceeded genome-wide threshold for plasma CysC as determined by permutation testing (Figure 1A). The CysC QTL on Chromosome 2 remained significant even after adjusting for the body weight as an additive covariate (Figure S3). We determined the 95% confidence interval by calculating the Bayesian Credible Interval (BCI), and identified a 1 Mb region of Chr 2, from 148.6-149.4 Mb associated with CysC.

**Figure 2:**
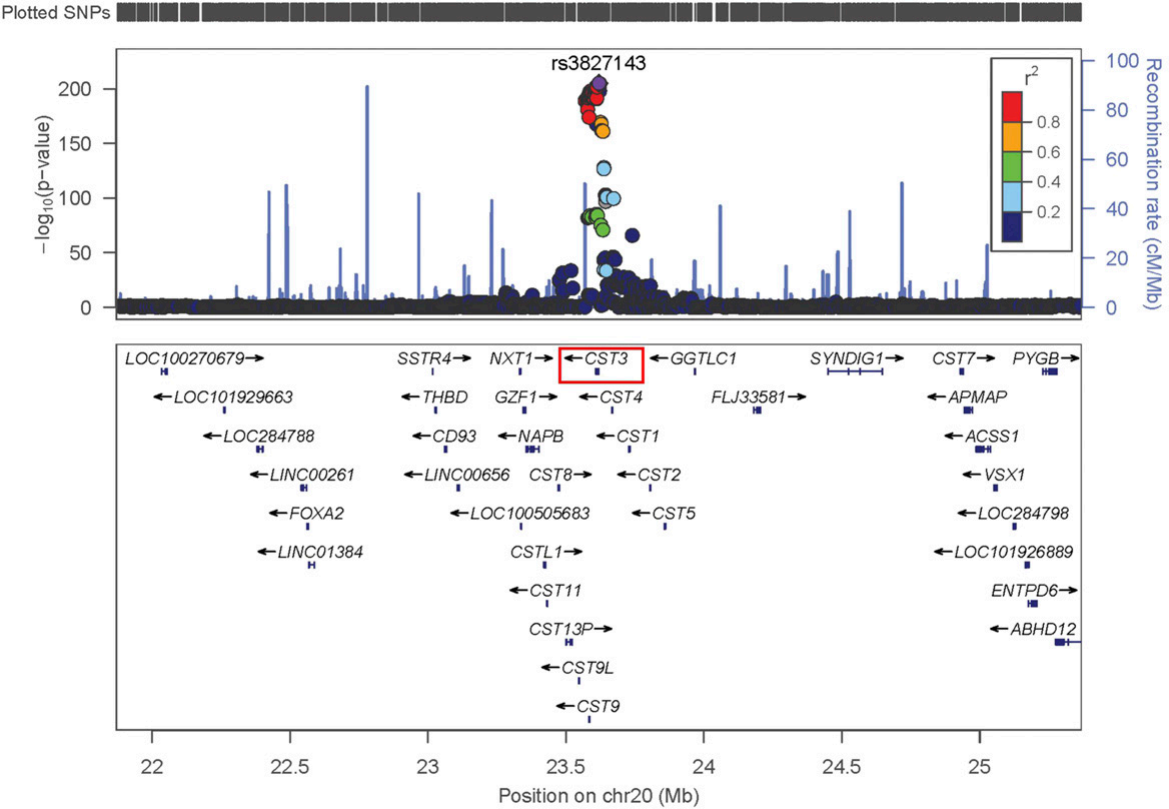
Locuszoom association plot for human eGFR-cys at the *Cst3* gene locus on human chromosome 20 between 22 Mb and 25 Mb. The human data were obtained from the European population (Pattaro *et al.* 2016) and is a region of conserved synteny between humans and mice.

Human GWAS studies have identified several loci associated with kidney disease and related physiological traits. Notably, the locus on Chr 2 for CysC in DO mice corresponds to the homologous locus in humans, on Chromosome 20, which has been replicated several times (Akerblom *et al.* 2014; Suhre *et al.* 2017; Köttgen *et al.* 2010) for measured human kidney function using CysC based estimates of glomerular filtration rate (eGFRcys). This association for human eGFRcys is near the *Cst3* gene locus on human Chr 20 between 22 Mb and 25 Mb in a European population (Pattaro *et al.* 2016) (Figure 2**)**, which is a region of conserved synteny between humans and mice.

**Figure 3:**
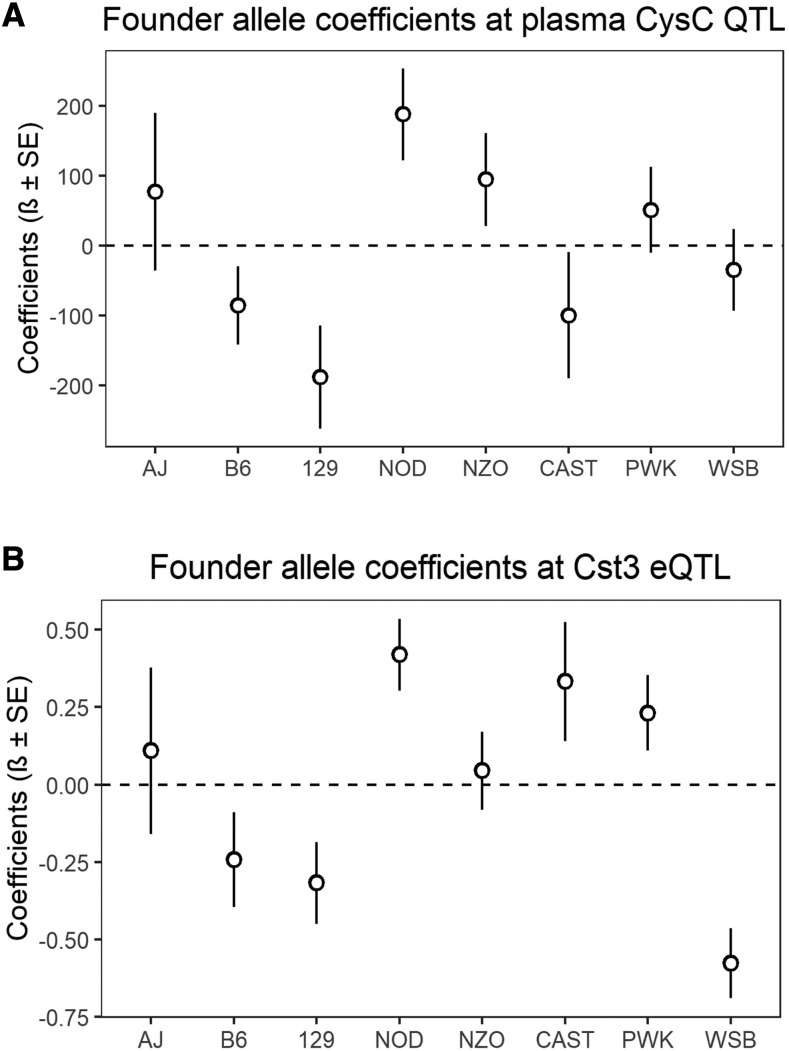
Regression coefficients of the association between genotype marker UNC4197823 located on Chr2 and (A) plasma CysC level or (B) Cst3 gene expression level for eight founder strain determined by BLUP analysis.

To further characterize this QTL, we estimated the allelic contributions of the 8 founder strains (Figure 1B and Figure 3A) and found that the haplotypes separate into two groups, in which, DO mice containing the 129, CAST, and B6 alleles have lower plasma CysC levels than mice harboring NOD and NZO haplotypes. We then determine the distribution of the founder allele among our DO mouse population and found that about 30.0%, 49.2%, 32.5%, 13.5%, 14.2%, 3.3%, 18.3% and 19.2% mouse are harboring genome from AJ, B6, 129, NOD, NOZ, CAST, PWK and WSB mouse, respectively either as homozygous or heterozygous state at the significant haplotype block of the CysC QTL (Table S2). This locus on the Chr 2 contains 32 pseudogenes and genes (Broman 2019; Yue *et al.* 2014) including *Cst3* (Cystatin C gene) (Figure 1C, Table S3). Among them, gene expression values of 16 genes were available and only *Cst3* mRNA expression was found to be significantly correlated (r = 0.52, adjusted p-value =6.57X10^−6^) with plasma CysC concentration (Table S3). Mice homozygous AA at the marker (UNC4197823, LOD = 10.3) had a lower plasma CysC level compared to heterozygous AG and homozygous GG (Figure 1D). To evaluate what other kidney gene expression levels might be associated with plasma CysC protein level we determined the correlation between plasma CysC level, and all genes expressed in kidney. Plasma CysC significantly correlated with a number of mRNA levels in kidney including *Cst3* (cystatin C; r = 0.52, adjusted p-value = 0.0048), *Trav16d-dv11* (T cell receptor alpha variable 16D-DV11; r = 0.52, adjusted p-value = 0.0048), *Pecam1* (Platelet/endothelial cell adhesion molecule 1; r = 0.50, adjusted p-value = 0.0064), and *Zfp768* (zinc finger protein 768; r=-0.45, adjusted p-value = 0.043; Table 2). We also performed QTL analysis of all other phenotypes listed on Table 1, which yielded a number of suggestive loci (Table S4).

**Table 2:** Genes significantly correlated with plasma Cystatin C, and their eQTLs

Gene name		ENTREZID	Gene location			Correlation			eQTL		
	Symbol		Chr	Stand	Start (Mb)	r	p	adj.p	SNP Position		LOD
	Chr	Mb
T cell receptor alpha variable 16D-DV11	*Trav16d-dv11*	547329	Chr14	+	54.20	0.522	2.98x10^−7^	0.005			
Cystatin C	*Cst3*	13010	Chr2	—	148.87	0.517	4.11x10^−7^	0.005	Chr2	148.7	15.99
Platelet/endothelial cell adhesion molecule 1	*Pecam1*	18613	Chr11	—	106.65	0.502	9.67x10^−7^	0.006	Chr6	136.7	9.50
Leupaxin	*Lpxn*	107321	Chr19	+	12.80	0.500	1.09x10^−6^	0.006			
FYVE, RhoGEF and PH domain containing 2	*Fgd2*	26382	Chr17	+	29.36	0.476	4.10x10^−6^	0.019			
Ribonuclease, RNase A family, 6	*Rnase6*	78416	Chr14	+	51.13	0.469	5.93x10^−6^	0.023			
Cytokine receptor-like factor 3	*Crlf3*	54394	Chr11	—	80.05	0.461	9.03x10^−6^	0.030			
Formin binding protein 1	*Fnbp1*	14269	Chr2	—	31.03	0.454	1.25x10^−5^	0.037			
Zinc finger protein 768	*Zfp768*	233890	Chr7	—	127.34	−0.449	1.62x10^−5^	0.043			
Protein kinase C, eta	*Prkch*	18755	Chr12	+	73.58	0.445	1.98x10^−5^	0.043			
Selectin, platelet (p-selectin) ligand	*Selplg*	20345	Chr5	—	113.82	0.445	1.99x10^−5^	0.043			

### Genome-wide expression QTL (eQTLs) analysis of Renal Gene Expression indicates CysC mRNA is under genetic regulation

**Figure 4:**
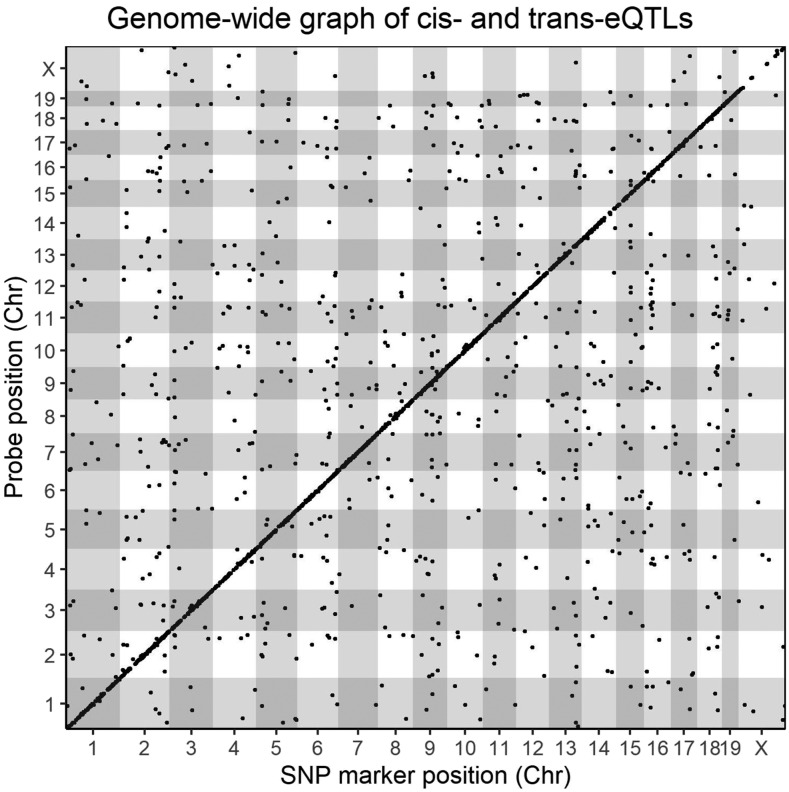
Locations of kidney eQTLs in the mouse genome. The positions of the significant eQTLs (*P* < 0.05) are plotted against the locations of the corresponding transcript (y-axis) along with the genome (x-axis). The significance thresholds for each individual probeset were determined by performing 1000 permutations of genome-wide scans by shuffling genotype data in relation to individual gene expression data for every single probeset on the microarray. *Cis*-eQTLs, occurring within a 4-Mb (±2Mb) genomic window, are located at the diagonal, all other dots represent *trans*-regulated genes.

We performed individual QTL analysis for all annotated probesets available on the microarray to identify genes with significant eQTL (Methods). We determined the significant eQTL based on single p-value cut off (*P* < 0.05), allowing for a unique LOD threshold for each individual probeset as determined by permutation testing (Methods), and observed a total of 3,022 significant eQTL hits (Figure 4) for 2,866 unique transcripts. The average genome-wide LOD threshold at *P* < 0.05 for all annotated probesets was 7.94 ± 0.67, with a median of 7.79 (25^th^ percentile= 7.69, 75^th^ percentile= 7.93), and a range 6.96– 21.27. Among the total 3,022 statistically significant (*P* < 0.05) eQTL, a total of 2,004 were *cis*-eQTL (peak SNP within ±2Mb of the gene start position (Bennett *et al.* 2010)) and 1,018 were *trans*-eQTL (Figure 3). A list of all significant and suggestive eQTL and their corresponding LOD threshold are provided in Table S5.

**Figure 5:**
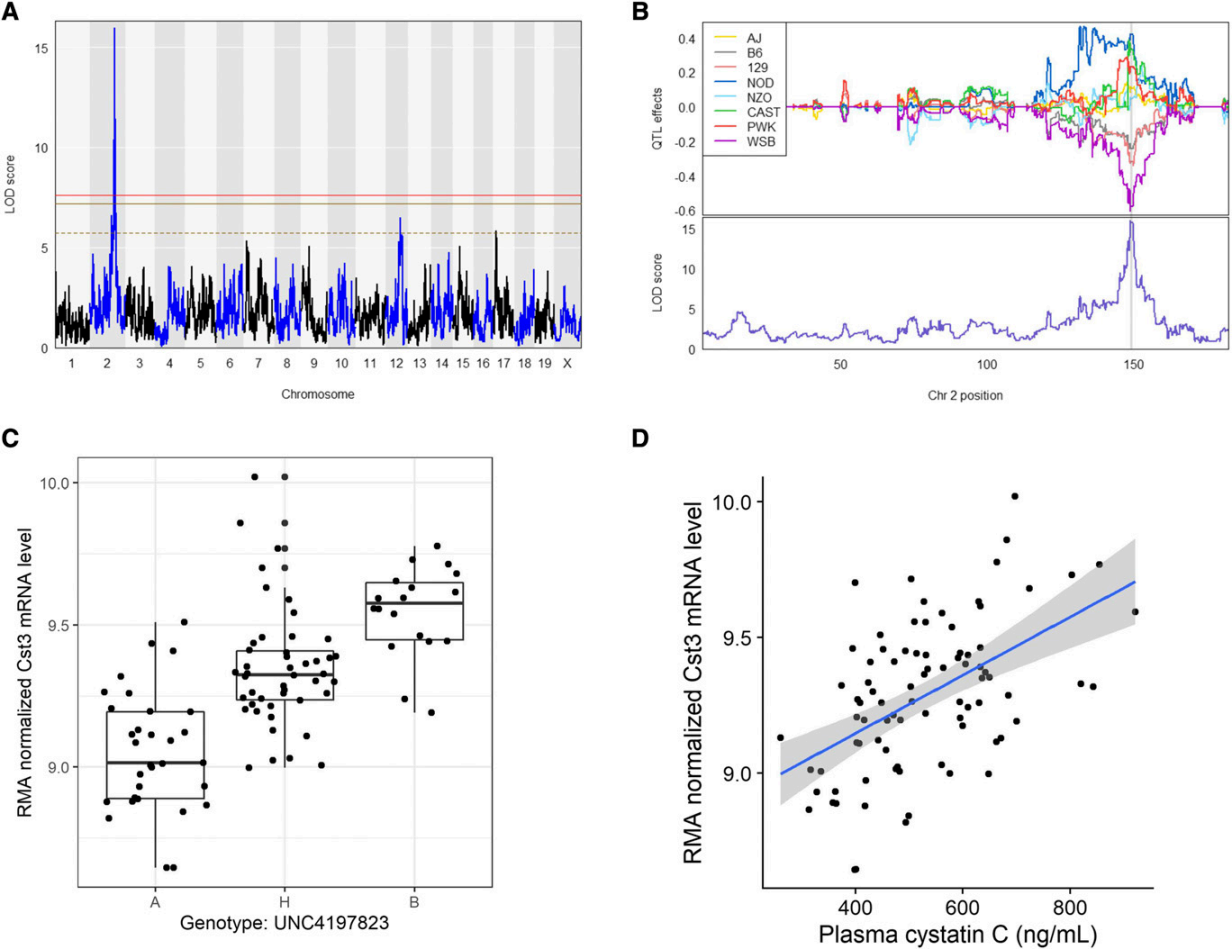
eQTL for Plasma Cystatin C (*Cst3*) mRNA. (A) Genome scan of CysC gene (*Cst3*) mRNA expression levels. Red, solid golden, and broken golden lines show permutation-derived (N = 1000) significance thresholds at *P* < 0.05, *P* < 0.10, and *P* < 0.63, respectively. (B) BLUP coefficient plot of eight founder mice strains to the *Cst3* eQTL (top). Color represents the eight founder mice strain as indicated. The bottom portion represents the LOD score for *Cst3* eQTL model on the Chr 2. The shaded area represents approximate 95% Bayesian credible interval. (C) Genotype by phenotype plot of the top SNP located at the peak LOD. Genotype: A – AA, B – GG and H – AG. (D) Correlation between plasma cystatin C and *Cst3* mRNA level.

We next investigated the expression pattern and genetic determination of genes at the CysC locus. We found that the mRNA levels of *Cst3* has a heritability of 58.8% and has a significant *cis*-eQTL (LOD = 16.0) at the same CysC locus on the Chr 2 (Figure 4A-C) with a similar association with the peak SNP indicating a possible connection between genetic architecture and plasma CysC level through mRNA levels. Indeed, in our study, plasma CysC concentration was associated with *Cts3* mRNA level [β (se) = 254.3 (45.6), *P* < 0.001] and remained significant even after adjusting with body weight [β (se) = 254.3 (46.0), *P* < 0.001] (Figure 5D). Mouse harboring the WSB, 129, and B6 alleles had lower Cst3 mRNA level compared to mice harboring CAST, PWK, NOD and NZO alleles (Figure 3B).

### Genetic variant analysis at the CysC locus

To better understand the genetic variation at the *Cst3* locus, we compared genetic polymorphism present in the 8 founder mouse genome available on the Sanger Institute’s mouse database (www.sanger.ac.uk) and found that there are several 5′ and 3′ UTR variations, splice region variants, upstream and downstream gene variant as SNPs or insertion or deletion in the DO founders, which may lead to the mRNA abundance. We took an *in silico* approach to predict transcription factor (TF) binding sites using CiiiDER (Gearing *et al.* 2019) with JASPAR 2020 motif database (Khan *et al.* 2018). This analysis predicted 426 potential TFs interacting to 2,657 TF motifs in the *Cst3* promotor region (-1500 bps to +500 bps of *Cst3* transcription starting position). We next identified which of the sequence variants from the DO founders are located within one of the TF binding sites. The 8 founder strains of the DO contain 31 SNPs located within these predicted transcription motifs. To assess the functional significance of these variants predicted changes in TF binding affinity was also assessed *in-silco* using the R package tRAP (Thomas-Chollier *et al.* 2011), which calculates the affinity of transcription factors for a DNA sequences on the basis of a biophysical model and determine which TF is affected the most by a regulatory SNP (Thomas-Chollier *et al.* 2011). tRAP predicted that 50 transcription motifs alter binding affinity at a level of >20% due to the presence of SNPs. A number of these SNPs including rs225697750, rs27261906, rs244335261, rs220753689, rs236432550, rs220753689, rs387212829, rs27261907, rs581036327, and rs236432550 were distributed differently among alleles arising from WSB and NOD, CAST, or PWK founder strains (Table S6).

We also examined the *Cst3* locus for structural variants using publicly available data in addition to variants affecting transcription (www.sanger.ac.uk). Among the 8 founder strains of the DO, two missense SNP variants in the *Cst3* gene are present in the CAST allele at position 148872018 bp and 148875196 bp (rs27261909). Both of these specific variants could potentially affect the protein function and the effect of these polymorphisms was assessed using an *in-silico* prediction tool, Protein Variation Effect Analyzer -PROVEAN (Choi and Chan 2015). However, the both missense variants in the *Cst3* gene were not predicted to have a known deleterious structural consequence (Table S7).

### Co-expression modules of kidney mRNA showed association Between CysC and biologically related gene sets

Our correlation/regression analysis identified that 24.8% of the variation in plasma CysC protein could be accounted for by differences in the *Cst3* gene expression. We hypothesized that additional genes and pathways affecting CysC levels could be identified using Weighted Gene Correlation Network Analysis (WGCNA). WGCNA can identify genetic pathways or modules of genes associated with clinical traits and provide a complementary approach to traditional QTL analysis. We constructed a gene co-expression network from 8,045 expressed genes in the kidney using WGCNA, as described in the method section. Out of these 8,045 genes, 4,166 transcripts formed 25 co-expressed gene modules, which contain a varying number of genes, ranging from 21 to 928 (Figure S4A-C). The remaining transcripts (∼3,900), had reduced topological overlap and were not assigned to a module.

**Figure 6:**
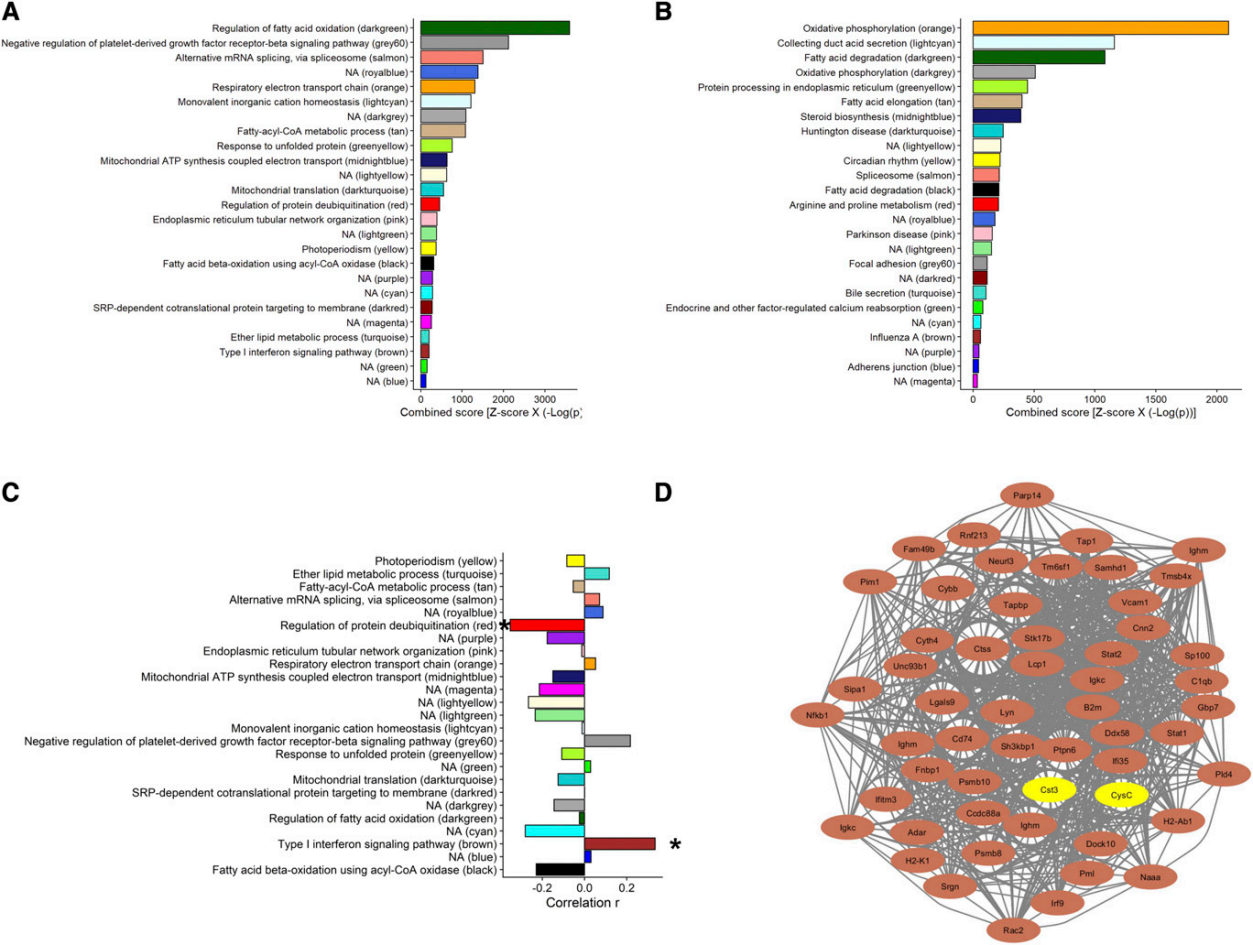
Co-expression network analysis of kidney gene. (A) Top GO and (B) top KEGG pathway for each of the gene-module. (C) Correlation coeffiencent of module eigengenes with plasma CysC. “*” < 0.05. (D) Cytoscape network visualization of to 50 hubgene in the brown module and their relationship with *Cst3* mRNA level is kidney and plasma CysC concentration. The brown nodes denote hubgenes in the module and the yellow nodes denote *Cst3* gene and CysC protein concentration in plasma.

To determine the correlation between gene modules and kidney biomarkers, we calculated the module eigengene (ME), which is the first principal component for the module member gene expression values. The average percent variance explained by the MEs was 27.0 ± 5.3% and ranged from 20.7% (greenyellow) to 39.0% (royalblue) (Table S8). In contrast, ME of the transcripts not assigned to a module explained 3.6% variability. Then we performed gene set enrichment analysis (Chen *et al.* 2013) for the GO and KEGG terms (Tables S9 and S10, respectively), as described in the method, on each module to determine their shared gene ontology of the biological function. 16 out of 25 modules were significantly enriched with biological process related GO terms (Figure 6A) and 18 were significantly enriched with KEGG pathway terms (Figure 6B).

The modules identified in the network were prioritized by their correlation with CysC (Figure 6C). Two modules, brown and red, showed significant positive (r = 0.33, adjusted p-value = 0.027) and negative (r = -0.35, adjusted p-value = 0.027) correlation with plasma CysC, respectively (Figure 6C). This indicates that the brown and red modules explain about 10.9% and 12.3% variation of the plasma CysC levels. The brown module contains 323 genes and is highly enriched for genes related to the immune system, specifically Type-I interferon signaling (GO:0060337, adjusted p-value = 1.05 x10^−5^, Odd Ratio =10.6, and combined score [Z-score x -log(p)] = 200.0 (Table 3). The 50 most connected transcripts within the brown module (Table S11) showed a strong correlation among themselves, *Cst3* gene, and CysC protein levels (Figure S5). We used Cytoscape network to visualize the connectivity among these 50 transcripts in the brown module and their connection with *Cst3* mRNA level in kidney and plasma CysC concentration (Figure 6D). Among these 50 transcripts are several well-characterized immune genes, including Signal transducer and activator of transcription 1 (*Stat1)*, Signal transducer and activator of transcription 2 *(Stat2)*, Interferon induced transmembrane protein 3 *(Ifitm3)*, Nuclear antigen sp100 (*Sp100)*, Interferon-induced protein 35 *(Ifi35)*, Sam domain and HD domain, 1 (*Samhd1*), and Interferon regulatory factor 9 *(Irf9)*.

**Table 3:** Enrichment analysis of the brown module genes

Term	P	adj.P	Odd Ratio	Combined Score *^a^*
Type I interferon signaling pathway (GO:0060337)	6.14 x 10^−9^	1.05 x 10^−5^	10.6	200.0
Regulation of interleukin-8 secretion (GO:2000482)	2.70 x 10^−5^	0.01	13.6	142.9
Positive regulation of response to cytokine stimulus (GO:0060760)	5.67 x 10^−5^	0.02	17.9	174.6
Response to interferon-alpha (GO:0035455)	1.30 x 10^−4^	0.03	14.7	131.6
Positive regulation of interleukin-6 secretion (GO:2000778)	1.30 x 10^−4^	0.03	14.7	131.6
Positive regulation of interferon-alpha production (GO:0032727)	2.06 x 10^−4^	0.04	13.2	111.7
Regulation of dendritic cell apoptotic process (GO:2000668)	4.48 x 10^−4^	0.05	18.8	144.6
Positive regulation of tumor necrosis factor secretion (GO:1904469)	6.09 x 10^−4^	0.07	17.0	126.2
Ribose phosphate metabolic process (GO:0019693)	8.02 x 10^−4^	0.08	15.6	111.4
Pentose-phosphate shunt (GO:0006098)	8.02 x 10^−4^	0.08	15.6	111.4
Response to chemokine (GO:1990868)	0.004	0.19	20.8	116.8
Cellular response to chemokine (GO:1990869)	0.004	0.19	20.8	116.8
Positive regulation of ATP biosynthetic process (GO:2001171)	0.004	0.18	20.8	116.8
Vesicle fusion with endoplasmic reticulum-Golgi intermediate compartment (ERGIC) membrane (GO:1990668)	0.004	0.18	20.8	116.8
Antigen processing and presentation of endogenous peptide antigen (GO:0002483)	0.004	0.18	20.8	116.8
Regulation of blood vessel remodelling (GO:0060312)	0.004	0.18	20.8	116.8
Regulation of interleukin-12 secretion (GO:2001182)	0.004	0.18	20.8	116.8
Regulation of interferon-alpha secretion (GO:1902739)	0.004	0.17	20.8	116.8
Positive regulation of interferon-alpha secretion (GO:1902741)	0.004	0.17	20.8	116.8
Glomerulus vasculature development (GO:0072012)	0.004	0.17	20.8	116.8

Results shows the top 20 gene ontology (GO) of biological processes based on combined score.

a The expected mean rank and standard deviation was calculated from running the Fisher exact test for many random gene sets for each term in the gene-set library and then the z-score was calculated to assess the deviation from the expected rank for the enriched GO term for the genes in the module. Finally, the combined score was computed by multiplying the z-score by the negative log of the p-value from the Fisher exact test.

Additionally, the red module was negatively associated with plasma CysC level (r =-0.340, adjusted p-value = 0.021). The red module contains 195 genes including D-amino acid oxidase (*Dao*), aldehyde dehydrogenase 2, mitochondrial (Aldh2), 4-hydroxy-2-oxoglutarate aldolase 1 (*Hoga1*), proline dehydrogenase (*Prodh*); proline dehydrogenase (oxidase) 2 (*Prodh2*), aldehyde dehydrogenase family 7, member A1 (*Aldh7a1*), and aldehyde dehydrogenase 9, subfamily A1 (*Aldh9a1*). KEGG pathway analysis revealed that the red module is enriched with arginine and proline metabolizing enzymes (adjusted p-value = 1.60 × 10^−4^, OR = 14.36, and combined Z-score = 207.6) (Table S10**)**. The red module is also significantly enriched with regulation of protein deubiquitination (GO:0090085, adjusted p-value= 0.026) with an OR of 44.0 and combined score of 456.3 (Table S9).

### Publicly available kidney gene expression datasets support association Between CysC and Type-I interferon signaling genes

In order to determine if the CysC-brown module association identified in the current study was robust we sought to identify appropriate publicly available datasets to investigate. We identified two gene expression data sets which utilize RNAseq, one utilizing C57BL/6J mice (EBI Array Express: E-MTAB-6081) as previously reported (Söllner *et al.* 2017) and one utilizing Diversity Outbred mice (GEO: GSE121330). We will refer to these as B6-RNAseq and DO-RNAseq. First, we ensured that the genes named within the manuscript are in fact expressed in the kidney. In supplemental Table S12 we list the expression of each gene mentioned in the manuscript and the two aforementioned datasets and found that they are expressed in the kidney. Additionally, we calculated the average expression for all genes in the datasets and listed this in the table along with the corresponding average expression for all eQTL genes and genes used in the Network analysis and found that they are comparable. Furthermore, we correlated the average expression values of the all common genes in our study, DO-RNAseq, and B6-RNAseq data sets and identified that broadly the genes selected are highly correlated (n = 16,599; r = 0.66; *P* < 0.001) and (n = 21,261; r = 0.60; *P* < 0.001), respectively). These data indicate that our measures of gene expression via microarray are representative of RNAseq methods and are reproducibly expressed in the kidney. We repeated this analysis with the 8,045 genes used for WGCNA analysis and these genes were also correlated between our data and renal samples of the DO-RNAseq (n = 7,818; r = 0.58; *P* < 0.001), and B6-RNAseq datasets (n = 7,825; r = 0.44; *P* < 0.001).

In addition to these global analyses, we also focused on the expression of brown module transcripts. We performed correlation analysis of the 50 most connected brown module transcripts and *Cst3* in the DO-RNAseq dataset. The correlation between these genes and *Cst3* was robust and ranged between r = 0.123 and 0.661. 48 of the selected transcripts were significant after multiple comparison testing (Figure S5). Most notable, the immune transcripts *Stat1*, *Stat2*, *Ifitm3*, *Sp100*, *Ifi35*, *Samhd1* and *Irf9* were all significantly correlated with *Cst3* which is congruent with the immune system enrichment identified in the brown module. As a final confirmation quantitative PCR assays were performed on archived kidney samples from a strain survey of DO Progenitor mice (O’Connor *et al.* 2014) in which the mice were perfused prior to tissue collection and these transcripts were expressed in perfused samples (Table S13).

## Discussion

Establishing the genetic architecture of kidney biomarkers remains critical to the development of clinical strategies for understanding kidney function. Advanced genetic mapping panels such as the Diversity Outbred (DO) mice provide a tremendous opportunity to examine the genetic determination of kidney function and disease. In this study, we utilize DO mice to dissect the genetic architecture of the renal biomarker CysC which yields 3 key results. The first is that CysC is associated with genetic polymorphism in aged, female DO mice. The second is that eQTL analysis identified a concordant eQTL for CysC mRNA, revealing a positive correlation between *Cst3* gene expression and plasma CysC levels. The third is two module of genes co-expressed associated with CysC levels. Each of these are discussed in detail below.

We identified a QTL associated with plasma concentration of CysC on Chromosome 2 at approximately 148 Mb. This locus contains the gene *Cst3* whose transcript is ultimately translated into the Cystatin C protein. eQTL analysis identified a significant *cis*-eQTL for the *Cst3* gene at the same locus and there was a positive correlation between *Cst3* mRNA and plasma CysC protein levels. Similar to our result, a meta-analysis of human GWAS data found that the estimated GFR based on plasma CysC was associated with SNPs proximal or within the physical location of *Cst3* on Chr20 at 23.6 Mb in human (Köttgen *et al.* 2010; Köttgen *et al.* 2009; Hwang *et al.* 2007). The identification of a colocalized eQTL for the *Cst3* gene and the correlation between transcript and protein levels in the current study suggest that a portion of the variable concentration of plasma CysC could be transcriptionally mediated. However, the potential causal variant(s) affecting mRNA levels remains to be elucidated. Detailed sequence analysis and utilization of *in-silico* prediction of the functional consequences of SNPs within the DO founder strains on transcription factor and protein function highlight the tremendous genetic variation contained in the DO at this locus. More than a hundred SNP variants were identified at the *Cst3* locus (Keane *et al.* 2011). Our *in-silico* analysis predicted 50 SNPs which could possibly affect *Cst3* mRNA levels and plasma CysC concentrations. However, these results remain to be confirmed.

The correlation between *Cst3* mRNA and plasma CysC suggests complex regulation of CysC beyond a simple relationship between mRNA levels and plasma CysC concentrations as only 24.8% of the variation in plasma CysC protein could be accounted by the variation in the *Cst3* gene expression. It is well understood that genetic variants are critically important factors affecting clinical traits, but we acknowledge that many complex traits are affected by a multitude of biological and environmental factors. One approach to address this biological complexity is the use of gene co-expression network analysis. Genes are often coregulated through complex biological pathways and co-expressed gene network analysis allows us to explore modulation of complex traits and disease phenotypes that are not understood by focusing on single genes. Therefore, we also identified a transcriptional networks (pathways) in the kidney associated with plasma CysC concentrations by using WGCNA analysis. Our gene module analysis showed that the plasma CysC level is highly co-expressed with genes involved in Type-I interferon (IFN) signaling pathway genes. Specifically, IFN-stimulated gene factor 3 (ISGF3), comprised of *Stat1*, *Stat2*, and *Irf9*, are strongly associated with CysC levels. To the best of our knowledge, this is the first report of local renal Type-I interferon signaling pathway gene expression being associated with transcriptional adjustment of CysC. The cell surface receptor for Type-I IFN are expressed on most cells, including kidney cells (Secombes and Zou 2017) and resident macrophages (York *et al.* 2007). When Type-I IFN binds with its receptor on the cell surface, a signaling cascade initiates, causing phosphorylation and activation of STATs (Schreiber 2017). We note that *in-vitro* experiments using a reporter assay identified a *cis* element named IRF (interferon regulatory factor)‐Ets composite sequence (IECS) that regulates both cystatin C and cathepsin C expression (Tamura *et al.* 2005). These data suggest a potential mechanism by which inflammatory signals contribute to plasma CysC levels. We note that our network analysis is non-directional and thus we cannot demonstrate a direct causal effect of IFN signaling and CysC gene expression. Furthermore, the relationship between CysC and Type-I IFN signaling genes is intriguing but complicated by the fact that CysC itself is expressed by monocytes and dendritic cells (El-Sukkari *et al.* 2003) and may have an important role in innate immune responses (Zi and Xu 2018). An additional study is needed to determine if this module of genes is regulating CysC or perhaps if CysC levels are influencing kidney inflammation. The *Cst3* gene expression only explained about 25% variation of the plasma CysC concentration, indicating that in addition to environmental factors, there might be some other genes or cluster of genes that contributing plasma CysC concentration.

Our results indicate that the DO mice can be used to study renal function in the context of varying genetic determination of *Cst3* levels, similar to what occurs in humans. Thus, perturbations that affect renal function through surgical or chemical manipulation could be used in DO mice to evaluate their effect on both renal function and CysC levels. Additionally, these studies are critical as studies in model organisms are effective at eliminating the random environmental factors. For example, studies in mice can tightly control the environment which allows the determination of the genetic contribution to the phenotype, independent of the environmental confounders.

The QTL, eQTL and WGCNA analysis identify a number of interesting factors affecting plasma CysC in this relatively small study utilizing DO mice. In addition to the sample size we acknowledge several areas for future investigation. Plasma CysC was measured only once at 56 weeks of age and thus we could not examine aging related changes in circulating CysC levels, which may be critical predictors of disease susceptibility. The DO cohort used in the current study is small (n = 120) and thus we were only able to detect QTL with strong effect size. We did not find QTL for kidney traits beyond plasma CysC and the *cis*-eQTL for *Cst3* which could be a consequence of our study population. Another limitation of our study is that we did not perfuse the kidney with PBS before collection. Thus, we cannot eliminate the possibility that differences in circulating white cells are contributing in part to gene expression differences observed in this study. To address this limitation and to confirm renal expression of the genes discussed in this paper, we compared our data with two different publicly available mouse kidney gene expression datasets. Additionally, the expression of a number of transcripts was confirmed by qPCR in kidney isolated from a set of DO progenitor mice which were perfused before tissue collection. Our expression studies are comprehensive but were performed on kidney tissue not specific cell types and thus provide limited insight into the specific kidney cell types mediating trait associations. Lastly, the results associating IFN signaling pathway gene expression with CysC levels is intriguing, but questions remain related to the temporal aspect of this association and how perturbation of genes in this module affect plasma CysC levels.

In conclusion, this study identified a locus on Chr 2 associated with variation in both plasma CysC concentration and the *Cst3* gene expression and a correlation between transcript levels of *Cst3* and plasma CysC. *In silico* sequence analysis highlighted a tremendous genetic variation contained in the DO population at this locus which potentially may affect transcript and protein levels, but a putative causal variant remained to be determined. Network analysis identified potentially novel inflammatory pathway associated with CysC concentration. Future DO mouse investigations are needed to explore the causal relationship between CysC, kidney inflammation and filtration function and their implications for CKD progression and adverse cardiovascular disease outcomes.
